# Whole genome sequencing and the application of a SNP panel reveal primary evolutionary lineages and genomic variation in the lion (*Panthera leo*)

**DOI:** 10.1186/s12864-022-08510-y

**Published:** 2022-04-22

**Authors:** L. D. Bertola, M. Vermaat, F. Lesilau, M. Chege, P. N. Tumenta, E. A. Sogbohossou, O. D. Schaap, H. Bauer, B. D. Patterson, P. A. White, H. H. de Iongh, J. F. J. Laros, K. Vrieling

**Affiliations:** 1 City University of New York, City College of New York, 160 Convent Avenue, New York, NY 10031 USA; 2 Institute of Environmental Sciences (CML), Leiden University, PO Box 9518, 2300 RA Leiden, The Netherlands; 3 Institute of Biology Leiden (IBL), Leiden University, PO Box 9505, 2300 RA Leiden, The Netherlands; 4 Department of Human Genetics, Leiden University Medical Center, 2300 RC Leiden, The Netherlands; 5 Leiden Genome Technology Center, Leiden University Medical Center, 2300 RC Leiden, The Netherlands; 6 Kenya Wildlife Service, Nairobi, Kenya; 7 Centre for Environment and Developmental Studies, Cameroon (CEDC), Yaounde, Cameroon; 8 Regional Training Centre Specialized in Agriculture, Forest and Wood, University of Dschang, BP 138, Yaounde, Cameroon; 9 Laboratoire d’Ecologie Appliquée, Université d’Abomey-Calavi, 03 BP 294, Cotonou, Benin; 10 Wildlife Conservation Research Unit, Zoology, University of Oxford Recanati-Kaplan Centre, Tubney, OX13 5QL UK; 11 Negaunee Integrative Research Center, Field Museum of Natural History, Chicago, IL 60605 USA; 12 Center for Tropical Research, Institute of the Environment and Sustainability, University of California, Los Angeles, CA 90095-1496 USA; 13 Department of Biology, Evolutionary Ecology Group, University of Antwerp, Groenenborgerlaan 171, 2020 Antwerpen, Belgium

**Keywords:** Single Nucleotide Polymorphism (SNP), Lion, Genomics, Genotyping, Evolutionary history, Genomic variation, Wildlife, Conservation, Africa, Biodiversity

## Abstract

**Background:**

Previous phylogeographic studies of the lion (*Panthera leo*) have improved our insight into the distribution of genetic variation, as well as a revised taxonomy which now recognizes a northern (*Panthera leo leo*) and a southern (*Panthera leo melanochaita*) subspecies. However, existing whole range phylogeographic studies on lions either consist of very limited numbers of samples, or are focused on mitochondrial DNA and/or a limited set of microsatellites. The geographic extent of genetic lineages and their phylogenetic relationships remain uncertain, clouded by massive sampling gaps, sex-biased dispersal and incomplete lineage sorting.

**Results:**

In this study we present results of low depth whole genome sequencing and subsequent variant calling in ten lions sampled throughout the geographic range, resulting in the discovery of >150,000 Single Nucleotide Polymorphisms (SNPs). Phylogenetic analyses revealed the same basal split between northern and southern populations, as well as four population clusters on a more local scale. Further, we designed a SNP panel, including 125 autosomal and 14 mitochondrial SNPs, which was tested on >200 lions from across their range. Results allow us to assign individuals to one of these four major clades (West & Central Africa, India, East Africa, or Southern Africa) and delineate these clades in more detail.

**Conclusions:**

The results presented here, particularly the validated SNP panel, have important applications, not only for studying populations on a local geographic scale, but also for tracing samples of unknown origin for forensic purposes, and for guiding conservation management of ex situ populations. Thus, these genomic resources not only contribute to our understanding of the evolutionary history of the lion, but may also play a crucial role in conservation efforts aimed at protecting the species in its full diversity.

**Supplementary Information:**

The online version contains supplementary material available at 10.1186/s12864-022-08510-y.

## Background

Recent developments in next generation sequencing (NGS) techniques allow for the application of massive parallel sequencing to non-model organisms [1, 2], such as the lion (*Panthera leo*). As a result, both the evolutionary history of a species and population histories can be reconstructed based on vastly expanded datasets [3, 4]. In addition to improved insight into the geographic distribution of genetic variation, this type of data can inform conservation efforts on how best to maintain this diversity [5, 6]. This is particularly relevant considering the dire situation of the lion populations in West and Central Africa, where downward populations trends are the strongest, and local extinctions have been reported in recent decades [7–11]. The IUCN Red List currently classifies the lion as ‘Vulnerable’ across its range, but states that it would actually meet the criteria for ‘Endangered’ in East and Central Africa, with lions in West Africa being.

‘Critically Endangered’ [12, 13]. Having a better understanding of intra-specific diversity in the lion can steer conservation efforts towards halting the loss of diversity.

The lion (*P. leo*) has been the subject of several phylogeographic studies which have provided insights into the evolution and distribution of genetic variation in the African populations (formerly subspecies *P. leo leo*) and its connection to the Indian population (formerly subspecies *P. leo persica*), located in and around the Gir National Park, Gujarat state, India. Although this Africa-Asia split has long been used to inform management (e.g. there is a separate studbook for Asiatic lions in zoos), this taxonomic distinction has since been overhauled. Phylogeographic studies played an important part in this, by providing improved understanding of the evolutionary history and relationships between populations. These studies included data from mitochondrial DNA (mtDNA) [14–23], autosomal DNA [18–20, 22–24] and subtype variation in lion Feline Immunodeficiency Virus (FIV_Ple_) [19]. Studies using mitochondrial markers and/or complete mitogenomes, describe a basal dichotomy, consisting of a northern group that includes populations from West and Central Africa as well as the Indian population (formerly recognized as a distinct subspecies), and a southern group with populations from East and Southern Africa [14, 16, 18, 21].

Two studies have included Single Nucleotide Polymorphisms, although both have included only two sampling localities in West and Central Africa and exact sampling localities were often unknown, hence calling for improved sampling in the region [22, 24]. The distinction of lions in West and Central Africa was further corroborated by autosomal microsatellite data [20]. Reliable inference of the evolutionary relationships between African and the Indian populations based on these data proved to be a challenge, as the extremely low variation in the Indian population led to an unresolved position in the distance tree [20].. Nevertheless, these newly described evolutionary relationships are reflected in a revised taxonomy which now recognizes a northern subspecies (*P. leo leo*) and a southern subspecies (*P. leo melanochaita*) [25].

However, each of the approaches used in the studies mentioned above have limitations. Although mtDNA is regarded as a useful genetic marker for gaining insight into phylogeographic patterns, partly because of its shorter coalescence time compared to nuclear markers, it may lead to ‘over-splitting’, reflecting fully coalesced groups based on mtDNA data, but incomplete lineage sorting of nuclear DNA (nuDNA) alleles. Moreover, mtDNA cannot identify admixture and represents only a single locus, potentially misrepresenting phylogenetic relationships due to the stochastic nature of the coalescent [26, 27]. In addition, sex-biased dispersal and gene flow will alter patterns derived from mitochondrial versus nuclear data. Because female lions exhibit strong philopatry whereas male lions are capable dispersers [28, 29], phylogeographic patterns based on mtDNA in lions may overestimate divergence between populations. Inference of phylogenetic relationships from microsatellite data, on the other hand, is problematic due to their high variability and their mutation pattern leading to homoplasy. Moreover microsatellite studies typically employ only a few dozen markers which effectively represents < 1% of the genome. Finally, most studies mentioned above were limited by sparse coverage of populations, notably in West and Central Africa. In particular, FIV_Ple_ prevalence is geographically restricted. Additional data from genome-wide markers, and covering more lion populations, are necessary to overcome these shortcomings. This will improve our understanding of the spatial distribution of variation in the lion, as well as help guide future conservation efforts that seek to preserve the species’ genetic diversity. As patterns of instra-specific variation are often shared across co-distributed taxa [21, 30], a deeper insight into these patterns of lion variation will also be relevant for other species.

Here, we describe the discovery and phylogeographic analysis of genome-wide SNPs based on whole genomes and complete mitogenomes of ten lions, providing an overview of the intraspecific genomic variation. We further developed a SNP panel consisting of a subset (*N* = 125) of the discovered SNPs, which was then used for genotyping > 200 samples from 14 countries, representing almost the entire current distribution of the lion. This resolves phylogeographic breaks at a finer spatial resolution, and may serve as a reference dataset for future studies. Finally, we discuss the applications and future directions of high-throughput genotyping for wildlife research and conservation with recommendations on how they may contribute to future studies on conservation genomics.

## Results

### Sequencing

The sequencing runs yielded a total of 6.5·10^8^ reads for all samples combined, corresponding to an average coverage of ~ 3.8 × per lion (see Supplemental Table 1 for coverage of autosomes and mitogenomes per individual). Following quality control, a total of 5.9·10^8^ reads (94.4%) were retained for subsequent alignment (Supplemental Table 1). More information on quality control is included in Supplemental Information 1 (and associated Supplemental Figures 1, 2, 3 and 4). Filtering of variable positions in lions with GATK yielded a total of 155,678 SNPs. Upon filtering for positions with coverage ≥ 3, we retained 118,270 SNPs of which 98,952 SNPs were called in at least five individuals (see Supplemental Table 2 for results for different levels of missing data). Missing data ranged from 90% (Benin) to 10% (Kenya) for all variable positions. Results reported for downstream phylogenetic analysis are based on the lion-specific variable positions which could be called in at least half of the included samples (98,952 SNPs).

### Whole genome data and complete mitogenomes

**Fig. 1:**
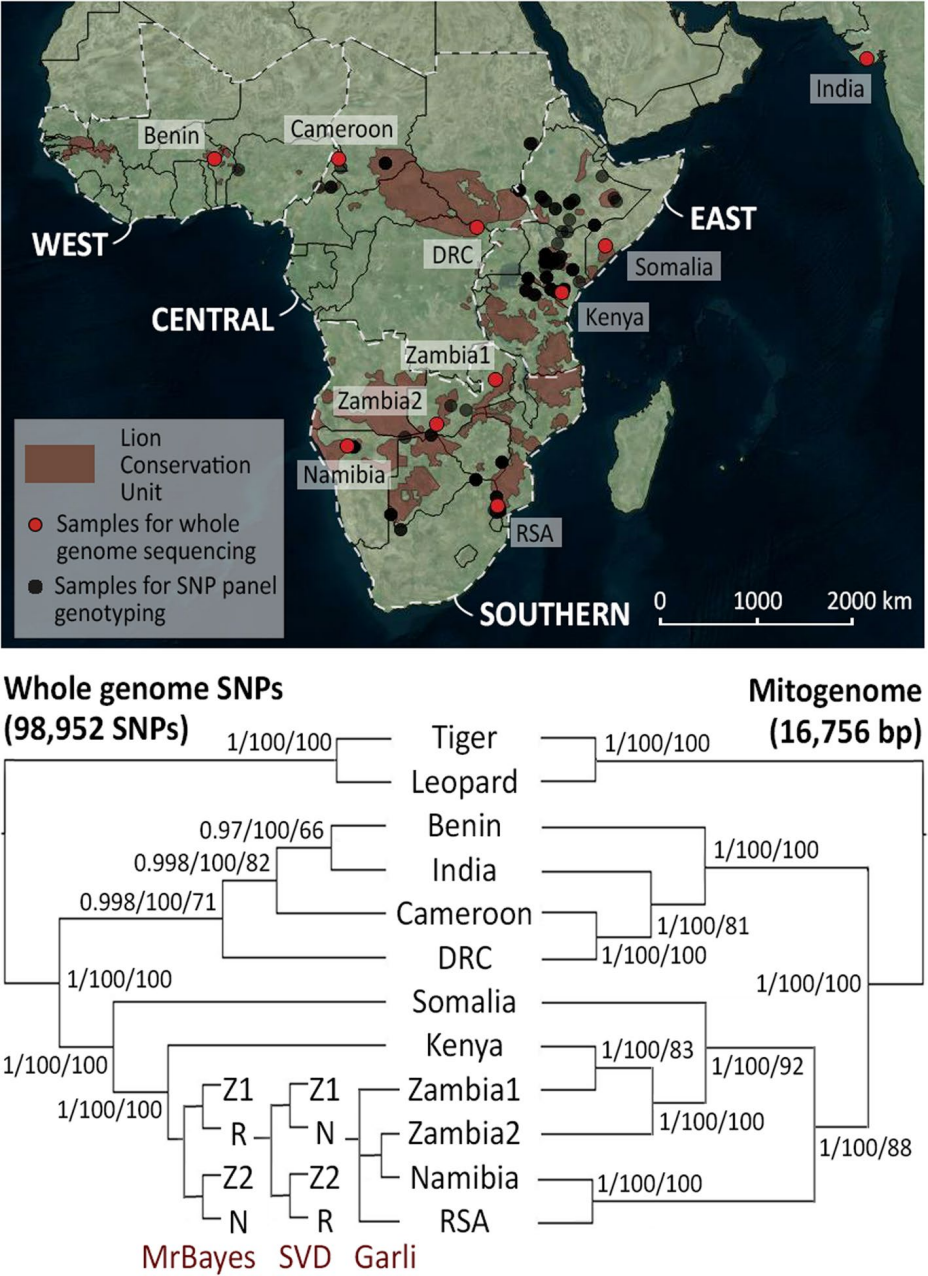
Distribution of lion sampling localities and inferred phylogenetic relationships between populations. Map indicating sampling locations of lions for whole genome sequencing (red) and SNP panel genotyping (black) (top). Red shading indicates Lion Conservation Units, and white delineation defines the regions (West, Central, East, and Southern), sensu the lion conservation strategies [31]. Phylogenetic trees, based on 98,952 nuclear SNPs (left) and 16,756 bp mitogenomes (right) for 10 lions which were subjected to whole genome sequencing (bottom). Support values indicate posterior probabilities (MrBayes), bootstrap support from SVDquartets and bootstrap support from Garli. Topologies are indicated per method in the southern branch of the autosomal SNP tree, and each split is maximally supported, with the exception of the Zambia2 + Namibia branch in the Garli tree, which received a bootstrap support of 96. Z1: Zambia1, Z2: Zambia2, N: Namibia, R: RSA. Base map from Bing Maps through OpenLayers plugin in QGIS 18.26

Phylogenetic analyses based on 98,952 SNPs, show a well-supported dichotomy between the northern populations (Benin, Cameroon, Demoratic Republic of the Congo (DRC), India) and the southern ones (Somalia, Kenya, Zambia1, Zambia2, Republic of South Africa (RSA), Namibia) (Fig. 1: left tree). Phylogenetic trees based on the mitochondrial genomes show the same basal dichotomy, with the Indian population nested within the northern populations (Fig. 1: right tree), as was previously reported [21]. However, it must be noted that the individual from RSA contains a haplotype from Namibia, likely the result of historic translocation, as was previously described [21]. MrBayes, Garli and SVDquartets resulted largely in the same topology, although the three methods do not agree on the relationships of Zambia1, Zambia2, RSA and Namibia based on the autosomal SNPs. We further compare results based on called genotypes and genotype likelihoods (Supplemental Information 2). A dendrogram based on genotype likelihoods shows the same basal dichotomy, including a split within the Southern subspecies (Supplemental Information 2, Supplemental Figure 5).

**Fig. 2:**
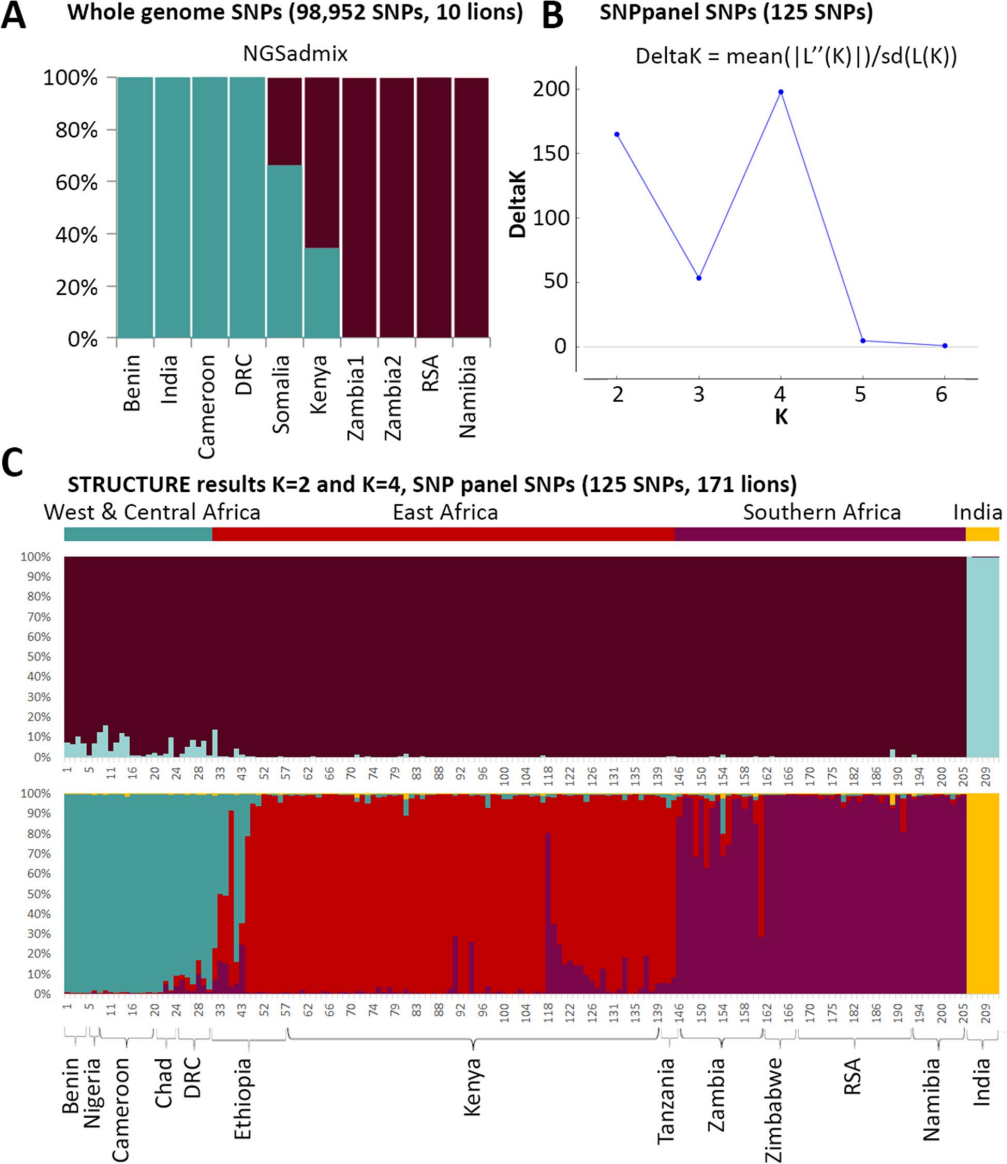
Assessment of population structure using NGSadmix and STRUCTURE. Results of NGSadmix (K = 2) based on SNP genotype likelihoods from 10 lions (A). Result of STRUCTURE Harvester for a STRUCTURE run of 125 SNPs genotyped (B). Assignment values based on the STRUCTURE run for K = 2 (upper bar plot) and K = 4 (lower bar plot) for 171 lions (i.e. excluding samples with > 25% missing data; see Supplemental Figure 6 for results for all 211 lions) (C). Sample numbers correspond with numbers in Supplemental Table 4

PCA based on genotype likelihoods and called genotypes show a comparable pattern, with a strong geographic signal (Supplemental Information 2, Supplemental Figure 6). Clustering of the ten individuals using NGSadmix and sNMF, identifies the same basal split into a northern and a southern cluster (Fig. 2A, sNMF results included in Supplemental Information 2, Supplemental Figure 7)). Based on the NGSadmix results, both Somalia and Kenya show signatures of admixture between the two subspecies. This is in line with results from the abbababa function in ANGSD. In the sNMF results, we observe a similar pattern for Somalia, but not for Kenya. This is further corroborated by the D-statistic (using GATK genotype calls) which resulted in significant admixture only for Somalia (but not for Kenya or DRC).

### SNP panel data

**Fig. 3:**
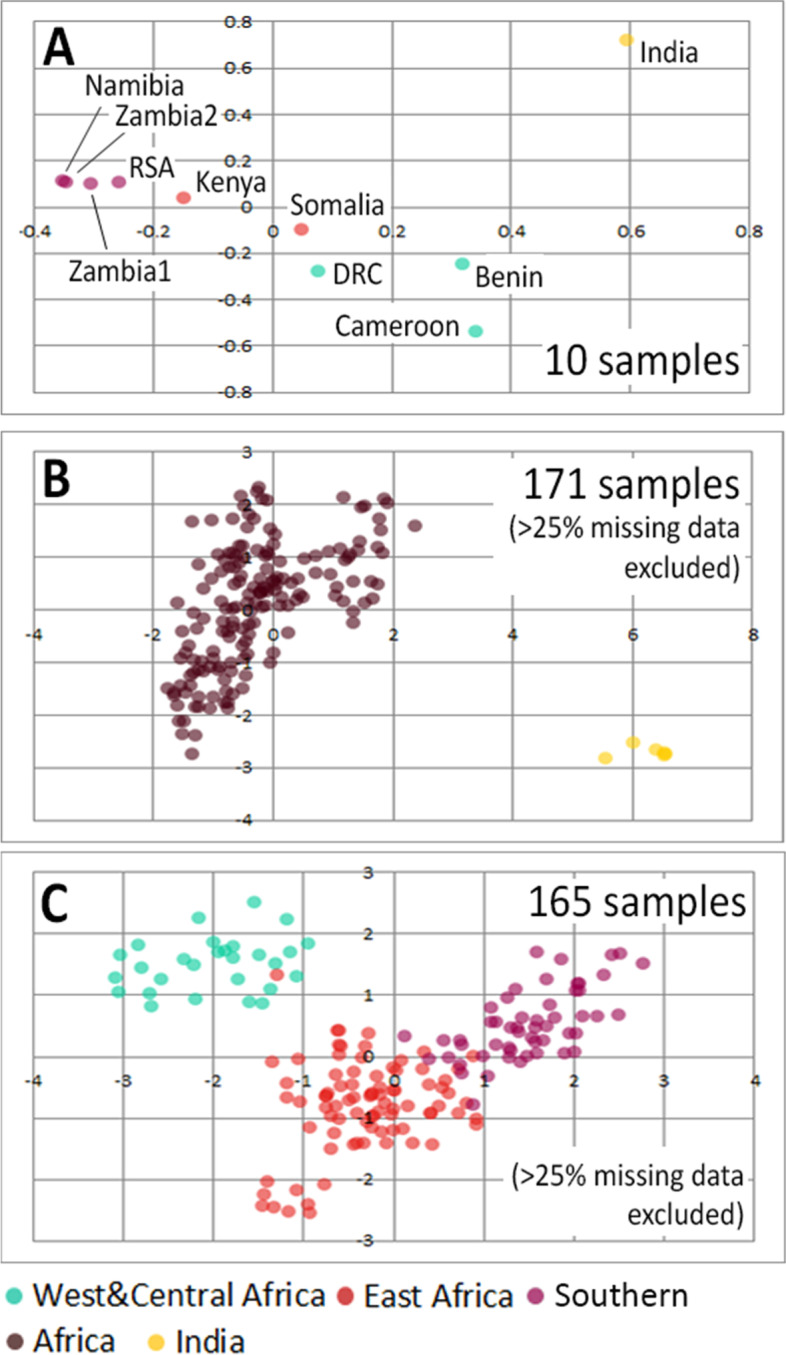
PCA plots based on whole genome genotype likelihoods and SNP panel SNPs. Plots are based on genotype likelihoods of 10 lions (A), 125 SNP panel SNPs in 171 lion samples (B), and after exclusion of India (C). Colours indicate region of origin

A total of 211 samples from across the entire range of the lion were genotyped for 125 autosomal SNPs and 14 mitochondrial SNPs. Missing data for the autosomal SNPs ranged from to 0 to 115 with a median value of 11; for the mitochondrial data, these ranged from 0 to 10 with a median value of 0 (Supplemental Table 4, Supplemental Figure 8, included in Supplemental Information 2). As a confirmation of our initial SNP calling, the individuals which were subjected to whole genome sequencing have also been included in genotyping through the SNP panel. No contradictory genotypes were found, corroborating the results from our initial SNP calling. STRUCTURE suggests an optimal number of four clusters (Fig. 2B) corresponding to West & Central Africa, India, East Africa, and Southern Africa (Fig. 2C: lower bar plot). A lower peak can be detected for K = 2, driven by India’s very low genetic diversity resulting in the formation of a strong separate cluster (Fig. 2C: upper bar plot). Because missing data can affect the results of a STRUCTURE run, we present here the results excluding individuals with > 25% missing data (*N* = 171). STRUCTURE plots including the entire lion dataset (*N* = 211) are available in Supplemental Figure 9 (included in Supplemental Information 2). Assignment values to clusters and to mitochondrial haplogroups are reported in Supplemental Table 5. In the PCA, African and Indian lions are distinguished as two clouds (Fig. 3B). Removing the Indian population reveals more structure within African lions (Fig. 3C), resulting in two nearly distinct clouds representing the northern (West & Central Africa) and the southern (East and Southern Africa) subspecies. PCA results based on whole genome genotype likelihoods are included as Fig. 3A, showing a congruent geographic differentiation compared to the SNP panel SNPs. PCA results for the entire datasets (i.e. including samples with > 25% missing data) are presented in Supplemental Information 2, Supplemental Figure 10.

**Fig. 4:**
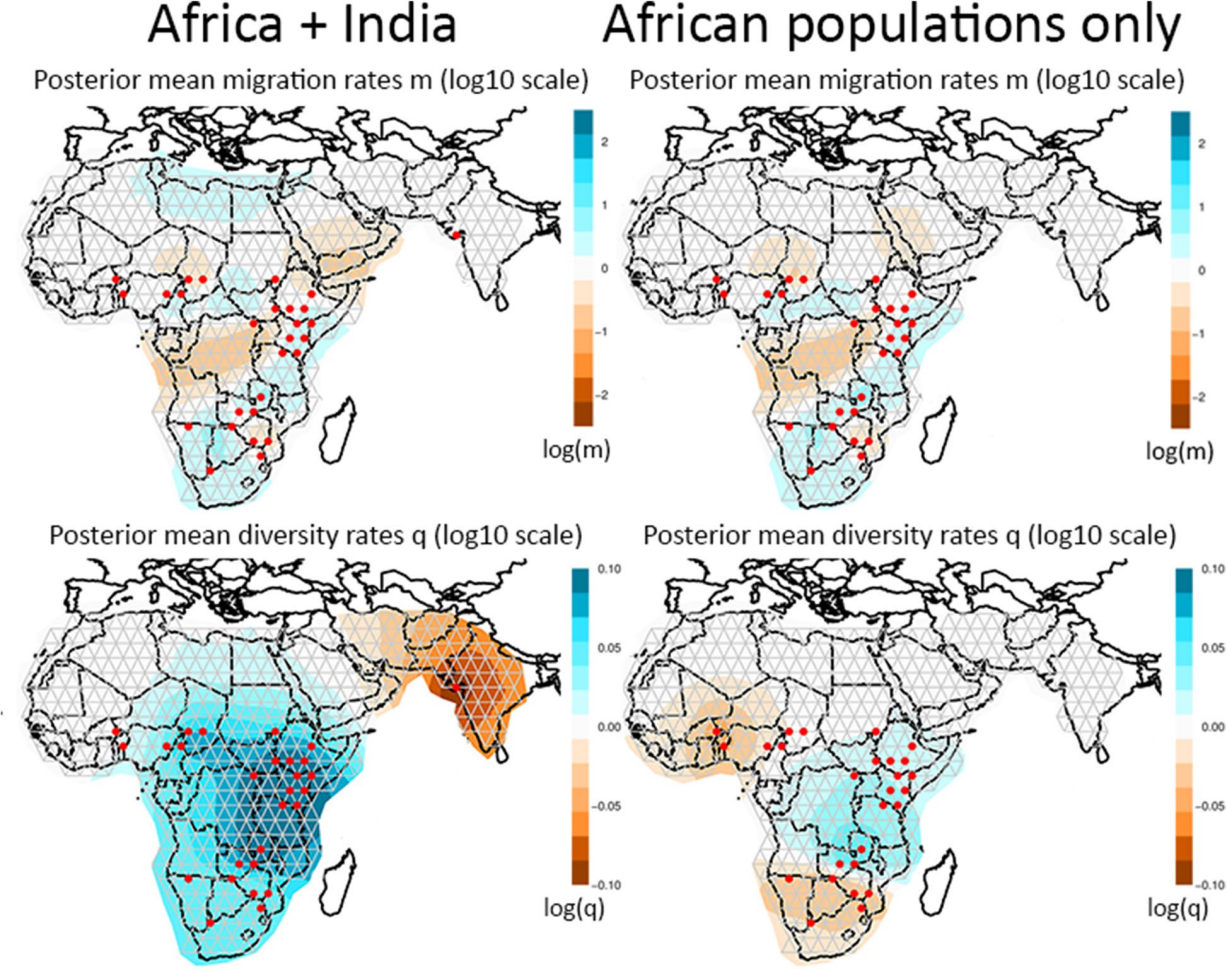
EEMS results based on 211 lions, including India (left), and 205 lions, excluding India (right). The upper row shows posterior mean migration rates, the bottom row shows posterior mean diversity rates. Orange colours indicate low values, blue colours indicate high values. Maps were generated with R code associated with EEMS

EEMS infers the existence of corridors and barriers for dispersal and gene flow from the spatial decay of genetic similarity. Results show that the Central African rainforest is highlighted as a barrier (indicated with orange shading, Fig. 4: upper row), whether or not the Indian population is included in the analysis. Barriers are further identified between East and Southern Africa, and across the Arabian peninsula. Diversity indices, which reflect the expected genetic dissimilarities of individuals sampled from the same deme, illustrate that the Indian population has a much lower genetic diversity than the African populations (Fig. 4: bottom left). After repeating the analysis with only African populations, low genetic diversity is detected in West Africa and Southern Africa (Fig. 4: bottom right).

### Heterozygosity

Comparisons of levels of heterozygosity across datasets and to previously published data [18, 20] (Supplemental Table 6) show that full genome observed heterozygosity is highly positively correlated with population-level observed heterozygosity derived from SNP panel SNPs and microsatellites (Supplemental Table 6: scatter plot and bar plot). The Indian population consistently shows a low observed heterozygosity for both SNPs and microsatellites (Supplemental Table 6: bar plot). However, for the estimates based on whole genome data, levels of heterozygosity are likely to be underestimated in the samples with low coverage (notably Benin).

## Discussion

### Whole genome data, complete mitogenomes and phylogeographic inference

Although SNPs can be powerful markers, ascertainment bias as a result of the study’s design is a concern [32]. To reflect the full diversity of the species, it is necessary to base the SNP discovery, for SNPs which can subsequently used in a SNP panel, on samples representing all lineages. Different types of markers used in lion phylogeographic studies show largely congruent results with some local discrepancies (e.g. widespread East/Southern Africa haplogroup not recovered from nuDNA data [20] and admixture in the Kruger area [21, 33]). Together, they provide useful criteria for selecting populations to be subjected to whole genome sequencing, as was done in this study.

Based on the whole genome data, we explored both genotype likelihoods, derived from ANGSD and genotype calls derived from GATK, as well as different levels of missing data, balancing the number of SNPs and the number of samples with an accepted call at a given position. As a higher number of SNPs represents a denser sampling of the coalescent (e.g. see [34]), presented trees are based on SNPs which were present in at least 50% of the samples. Increasing the number of SNPs (and therefore also the amount of missing data), did not change the topology or the support of the phylogenetic trees. Simulation studies and studies using empirical data have shown that concatenated SNP data are able to produce reliable trees reflecting the true topology, as long as enough genes are sampled [35, 36]. The underlying assumption is that there is enough phylogenetic signal in the data, and that discordant coalescent histories will effectively cancel each other out when all histories are considered together. The conclusion that our concatenated phylogenetic trees (MrBayes and Garli) produce reliable topologies is supported by the fact that observed patterns are congruent with results of SVDquartets, which does assume multi-locus unlinked data. An exception is the more fine-scale topology for Southern Africa where patterns of differentiation are likely to be affected by continuous gene flow and incomplete lineage sorting. Further, described topologies are in line with previously described mitochondrial trees [14, 21] with similar discordances as found in microsatellite datasets [20]. Notably, the wide-spread haplogroup labeled as East/Southern Africa [21], stretching from Kenya to Namibia, is not recovered from microsatellite [20] or SNP data (this study). The previously mentioned dichotomy between the northern subspecies, *P. leo leo*, and the southern subspecies *P. leo melanochaita*, is also supported by the assignment values of NGSadmix and sNMF, with possible admixture where both subspecies overlap.

### SNP panel data

Based on the ten whole genomes, we generated a SNP panel of 125 autosomal and 14 mitochondrial SNPs which allows cost-effective genotyping of larger numbers of samples.

STRUCTURE results from the SNP panel data from lions across their natural range identify four clusters: West & Central Africa, India (the two clades of the northern subspecies, *P. leo leo*), East Africa, Southern Africa (the two clades of the southern subspecies, *P. leo melanochaita*) (Fig. 2C). Main regions of admixture are Ethiopia and Zambia, as is visible in the STRUCTURE plots (Fig. 2C), and was also found using microsatellite data [18, 20] and mtDNA [37]. It is well known that STRUCTURE is sensitive to groups of closely related individuals, such as siblings, family groups, or in our case, an inbred population [38, 39] and that identification of ancestral populations may be an over-interpretation of the data, depending on demographic histories [40]. Here, the West and Central African populations show an ancestral relationship to the Indian cluster, while also harbouring higher genetic diversity, leading to similarity to the southern subspecies cluster. The PCA results further illustrate that variation between African populations is masked by the extremely low diversity in the Indian population, likely the result of isolation and small population size due to multiple bottlenecks and subsequent genetic drift (also see results on heterozygosity, Supplemental Table 6). The variation between African populations only becomes apparent when exploring a higher number of clusuters (i.e., K = 4; Fig. 2C, bottom plot) or excluding the Indian populations (Fig. 3C). After excluding the Indian population in the PCA, three overlapping clouds are apparent corresponding to West & Central Africa, East Africa and Southern Africa (Fig. 3C). The individual which has a color code from East Africa (red) but falls in the West and Central Africa cluster (green) originates from Ethiopia, where both subspecies are known to overlap and admixture has been previously described [20, 21]. The distinction between East and Southern Africa seems to be more gradual in PCA space, which is in line with the widespread haplogroup that occurs throughout almost the entire region.

### Evolutionary history and connectivity

In order to put the phylogenetic patterns of the lion into an evolutionary perspective, it is worthwhile to explore current and historical barriers to lion dispersal. Paleoclimatic data show that cyclical contraction and expansion of vegetation zones, e.g. rain forest and desert, may have acted as temporal barriers to lion dispersal [21]. The discrete genetic lineages recognizable in the mtDNA are likely to be the result of the restriction of suitable lion habitat to a number of refugia [21]. The pattern found in mtDNA data of the lion is congruent with that of other African savanna species [21, 30, 41] and predicted refugial areas based on climate models [42]. Faster coalescence times of mtDNA may have led to reciprocally monophyletic mtDNA clades in the lion, while isolation in refugia may not have lasted long enough for coalescence in autosomal markers [20, 21]. In addition, dispersal in lions is male-biased [28, 29], which may explain the more discrete phylogeographic pattern found in mtDNA data. This is reflected by the fact that we do not retrieve a North East Africa cluster or a South West Africa cluster based on autosomal SNPs, even though they are represented by diverged mitochondrial haplotypes. Interestingly, the discrepancy in population structure between autosomal and mitochondrial markers in East and Southern Africa, where a wide-spread mitochondrial haplogroup occurs from Kenya to Namibia and autosomal markers suggest a phylogeographic break around Zambia and Mozambique, is identical to the discrepancy found in giraffe [43]. Current barriers for gene flow seem to be mainly represented by the recent population disjunction in North Africa/Middle East and the longstanding barrier representing the Central African rain forest, as is also inferred by EEMS (Fig. 4). Although the Rift valley, stretching from Ethiopia in the north to Malawi and Mozambique in the south, has been mentioned as a potential barrier for gene flow in the lion (Dubach et al*.* 2005; Barnett et al*.* 2006a; Barnett et al*.* 2006b; Bertola et al*.* 2011) and gene flow may be reduced in that region, co-occurrence of strongly diverged haplotypes and admixture detected in microsatellite and SNP data in Ethiopia indicates that the Rift valley does not represent an impenetrable barrier for lion dispersal [20, 21].

### Applications for wildlife research and conservation

The design of a SNP panel and a reference dataset of > 200 lions from 14 lion range states has important applications for wildlife research and conservation. First, it allows us to distinguish phylogeographic breaks and overlap between lineages which can be used to study the evolutionary history of the species in more detail, e.g. at the national level. This may have important conservation implications. For example, genomic data can be used to prioritize lion management at the population level, including providing key information for decisions regarding translocations of individuals within and among lion range states. Based on CITES documentation, > 1000 live lions have been imported into lion range states since the 1980s with (future) potential to interbreed with resident lions (Bertola et al*.*, https://onlinelibrary.wiley.com/doi/full/10.1111/eva.13318). Human-mediated movement of wildlife, regarding lions and other species, only rarely take genetic considerations into account. Providing a baseline of the distribution of genetic variation and a tool to screen potential candidates for translocations, can help support these initiatives with scientific data. To assess the suitability of this SNP panel on a smaller geographic scale, we are currently genotyping > 200 lion samples from across Kenya. The aim is to provide a robust tool for wildlife managers seeking genetic support for management decisions. Secondly, it enables tracing of lion samples of unknown origin, such as material confiscated from illegal trade chains or through anti-poaching efforts. Illegal trade in wildlife products is currently estimated to be the fifth largest illegal industry globally, and a major concern for conservationists [44–46]. Part of the trade is thought to cater to domestic markets, but growing evidence suggests an increase in illegal shipments to international markets [47]. Genetic toolkits can contribute to combatting wildlife crimes by identifying confiscated material, source populations and tracking trade routes [33, 48–51]. For lions, a tool that assists law enforcement intelligence has already been designed using mtDNA haplotypes (lionlocalizer.org), and it offers an ideal opportunity to include autosomal SNPs in future updates of this tool. Thirdly, a SNP panel can be used to guide breeding efforts for ex situ conservation [33, 52]. Recently, our SNP panel was used to genotype a batch of lion samples from institutions linked to the European Association for Zoos and Aquaria (EAZA). Based on the resulting information, managers are currently deciding how many and which lineages to include in future breeding efforts. In particular, given the dire situation for lions in West and Central Africa, and a severe under-representation of this lineage in captivity, the use of SNP data can be helpful to efficiently identify and conserve diversity ex situ and prevent inbreeding.

### Future perspectives

With the increase of genomic data alongside computational and technological developments, the field of conservation genomics is undergoing a major transition. Demographic histories and patterns of gene flow can be inferred in greater detail [53, 54]. Searching genomes for signatures of adaptation and deleterious alleles has been highlighted as a powerful tool for gauging the sensitivity of populations in a changing environment [55–59]. New developments allowing SNP genotyping from poor quality (e.g., non-invasively collected) samples will further contribute to the applicability of SNP genotyping in the field [60]. And, finally, the development of mobile, hand-held sequencing devices shows great promises for rapid, real-time identification of samples [61]. Such tools represent tremendous potential for training and capacity building [62] especially for biodiverse countries with limited facilities to process samples, thereby making the field of wildlife research and conservation more democratized and inclusive.

## Conclusions

This study is the first to report phylogeographic relationships between lion populations throughout their entire range based on ~ 150,000 SNPs derived from whole genome sequencing. We present a SNP panel, containing 125 autosomal and 14 mitochondrial SNPs which has been validated on > 200 individuals from 14 lion range states, spanning most of the lion’s range. The results reveal a detailed population structure and confirm a basal distinction between a northern and a southern subspecies that supports the recent revision of the lion’s taxonomy. We highlight several applications for the genomic resources we present here, and the SNP panel in particular. The samples on which the panel was tested can serve as a reference database for future research and conservation efforts.

## Methods

### Sampling

Blood or tissue samples of ten lions, representing the main phylogeographic groups as identified in previous studies [14, 16, 19–21] (Fig. 1: map, Supplemental Table 1), had been collected in the context of previous studies by this research group (see [21]) and preserved in a buffer solution (0.15 M NaCl, 0.05 M Tris–HCl, 0.001 M EDTA, pH = 7.5) at -20 °C. Individuals were free-ranging lions from the following localities: Benin (Pendjari NP), Cameroon (Waza NP), DRC (Garamba NP), Kenya (Tsavo East NP), Zambia (Luangwa Valley and Mulobezi Town), and Namibia (Etosha NP). The individual representing India was captive born in Sakkarbaug Zoological Garden (Junagadh, Gujarat, India), from two wild born parents from the Gir forest, India. The individual from Somalia was a confiscated individual, housed at Safaripark Beekse Bergen (Hilvarenbeek, The Netherlands), and the individual from RSA was obtained from Ouwehands dierenpark (Rhenen, The Netherlands) with proper documentation of their breeding history (see Supplemental Table 1 for details on the origin of all individuals). A sample from an Amur leopard (*P. pardus orientalis*, captive), obtained from Planckendael Zoo (Muizen, Belgium), was included as an outgroup. The Amur tiger (*P. tigris altaica*) genome (816 scaffolds: Genbank Accession numbers KE721553-KE722368) [63], supplemented with a high quality lion mitogenome (Genbank Accession number: KP001493) [21], was used as a reference for mapping of the lion and leopard reads. All samples were collected in full compliance with specific legally required permits (CITES and permits related to national legislation in the countries of origin). Details on laboratory protocols, sequencing, mapping to the reference genome, SNP calling, and quality control are given in Supplemental Information 1 (including Supplemental Figures 1, 2 and 3, and referring to Supplemental Table 7 and 8).

### SNP calling and complete mitogenomes

After mapping of the reads to the reference genome (tiger reference genome from [63] and lion mitogenome from [21]), autosomal SNPs were called using two methods: 1) we used ANGSD [64] to derive genotype likelihoods, and 2) we called and filtered SNPs using GATK [65] and VCFtools [66] (details in Supplemental Information 1). Because these results stem from low coverage genomes, and acknowledging the potential confounding effect of missing data, we explored different levels of missing data applying the –minInd flag in ANGSD. For the SNPs derived from GATK we replaced positions with coverage < 3 by an ambiguous nucleotide, and we applied 5 levels of filtering as follows: 1) all SNPs, 2) SNPs called in at least three samples, 3) SNPs called in at least five samples, 4) SNPs called in at least eight samples, and 5) SNPs called in all samples (Supplemental Table 2). Identified SNPs were attributed to a chromosome following the genomic architecture in the tiger [63] (Supplemental Table 2). Full mitogenomes were recovered by mapping all reads to a mitogenome reference generated with long range PCR and a known numt sequence, allowing us to differentiate between reads of mitochondrial and nuclear origin. This procedure is described in more detail in Supplemental Information 1 and [21].

Phylogenetic analyses were performed on the full mitogenomes and on the concatenated SNP datasets with varying levels of missing data with MrBayes v.3.1.2 [67, 68] and Garli [69] using parameters as determined by MrModeltest2 (v.2.3) [70]. MrBayes and Garli were run for one million generations and five million generations respectively, using a GTR substitution model with rate variation across sites set to equal. In addition, we ran SVDquartets [71], assuming multi-locus unlinked single-site data with 100 bootstrap replicates as implemented in PAUP* 4.0a164 [72]. For the mitogenome data, the coalescent process in the model was disregarded. Nodes receiving > 95% Posterior Probability (PP) in Bayesian analysis (MrBayes) and 0.7 bootstrap support in Maximum Likelihood (ML) analysis (Garli) and SVDquartets are considered to have significant support, as is common practice. A dendrogram was created on the genotype likelihoods derived from ANGSD, using the hclust algorithm. Genotype likelihoods (ANGSD) and genotype calls (GATK) were further subjected to Principal Component Analysis (PCA), using PCAngsd [73] and the PCA function from the Adegenet R package [74], respectively. Individual ancestry coefficients were estimated using NGSadmix [75] on the genotype likelihoods. For GATK genotypes, we used sparse non-negative matrix factorization algorithms in sNMF, as implemented in the R package LEA [76, 77], exploring K = 1–10, using 20 replicates and 4 values for the alpha regularization parameter (1, 10, 100 and 1,000). To explore putative admixture, we used the abbababa function in ANGSD and we calculated D-statistics (ABBA-BABA test) on the GATK genotypes using CalcD from the evobiR package [78] with 1000 bootstraps. We used Cameroon and Zambia1 as the ingroup as they are clearly assigned to *P. leo leo* and *P. leo melanochaita* respectively, and tiger as the outgroup.

### SNP panel data

In order to obtain better insight into the geographic locations of phylogeographic breaks, we made a selection of SNPs for inclusion in a SNP panel for genotyping more samples. We used the following criteria for the selection process: 1) minimum coverage of 20 for all lion samples combined, across 50 bp upstream and downstream of the SNP position, 2) maximum of one variable position in these 50 bp flanking regions, 3) high quality mapping of the flanking regions as identified by eye using IGV Genome Browser [79, 80], 4) at least eight individuals represented at each selected site, 5) SNPs evenly spread across all chromosomes, as implied by the chromosomal architecture in tiger (max. one SNP per scaffold), 6) preferably both homozygotes and heterozygote genotype present among the ten genotyped lions (but sites for which each individual was scored as a heterozygote were excluded, as these may represent gene duplications). In addition, we included a total of 14 mitochondrial SNPs selected to represent each major branch within the mitochondrial phylogenetic tree (as described in [21]), as well as two diagnostic SNPs that represent the distinction between the northern and the southern subspecies. These mtDNA SNPs had already been assessed in a wide range of populations [21], making it more likely that the selected SNPs are diagnostic throughout the lion range. Finally, we ensured that the mitochondrial SNPs were not located in any of the nuclear copies (numts) which are known to exist in cats [81–85] by aligning our mitogenome sequences with known numt sequences. Coordinates of each of the SNPs and its associated chromosome, both for the tiger genome [63] and for a nearly-chromosome level lion genome [86] (lion genome was not available when the original mapping was done) are reported in Supplemental Table 3 and visualized in Supplemental Figure 4 (included in Supplemental Information 1). After test runs and quality control (see Supplemental Information 1), we retained 125 autosomal SNPs and 14 mtDNA SNPs which were used for genotyping 211 lions of known origin from 14 lion range states representing the entire geographic range of the species (Fig. 1: map). There is a special focus on the region where the ranges of the northern and southern subspecies overlap i.e., Ethiopia and Kenya. This region is known to harbor haplotypes from strongly diverged lineages [21], and microsatellite data have suggested admixture [20]. SNP genotyping was done at the SNP genotyping facility at Leiden University using allele-specific primers and KASP technology (LGC Genomics).

Resulting autosomal genotypes were analysed with STRUCTURE [87] using correlated allele frequencies and running the program for 5 million generations, discarding the first 500,000 generations as burn-in. Five replicates were run for K = 1 to K = 7. The optimal number of K was assessed by using the DeltaK method as implemented in STRUCTURE Harvester [88, 89]; CLUMPP [90] was used before generating the bar plots of population assignment. To reduce the effect of missing data on the assignment results, we ran STRUCTURE using the complete dataset (*N* = 211) and a dataset excluding samples with > 25% missing data (*N* = 171). MtDNA SNPs were used to assign a specific haplotype to each individual, matching with previously described lineages (see delineation of haplogroups in [21]): West Africa, Central Africa, North East Africa, East/Southern Africa, South West Africa, and India.

A PCA was performed in Genalex [91], using the entire dataset (*N* = 211) and the reduced dataset excluding samples with > 25% missing data (*N* = 177). Because the PCA is disproportionately affected by the very low genetic diversity of Indian lions, we repeatedthe analysis excluding this population, thereby allowing a more detailed picture from the African populations to emerge.

Patterns of connective zones and barriers were investigated using Estimated Effective Migration Surfaces (EEMS) [92, 93] for all individuals genotyped with the SNP panel. Three independent runs were performed, using 10 million generations and discarding the first 5 million generations as burn-in. We followed the authors’ suggestions for tuning the proposal variances, using 0.1 and 1 for mSeedsProposalS2 and mEffctProposalS2 respectively, and 1.5 and 0.015 for qSeedsProposalS2 and qEffctProposalS2 respectively.

### Heterozygosity

The level of observed heterozygosity was assessed for each individual for which the whole genome had been sequenced, expressed as the proportion of heterozygote positions compared to the total number of SNPs, excluding positions with ambiguous nucleotides (i.e. positions which were not scored due to insufficient quality or coverage). This was done on the individual level for all SNPs, and on the populations level for all SNP panel SNPs which were successfully called in five or more individuals from the same population. Results were then compared to known levels of heterozygosity based on earlier studies using microsatellite data from the same populations [18, 20].

## Supplementary Information

**Additional file 1:** **Supplemental Information S1.** Details on laboratory protocols, sequencing, mapping, SNP calling and quality control (including Supplemental Figures S1-S4).

**Additional file 2:** **Supplemental Information S2.** Validation of phylogenetic inferences and populations structure from whole genome sequencing and SNP panel data (including Supplemental Figures S5-S10).

**Additional file 3:** **Supplemental Table S1.** Lion and leopard samples included for whole genome sequencing and results from sequencing runs. **Supplemental Table S2.** Number of discovered lion SNPs per chromosome and estimated chromosome size in tiger (derived from Cho et al. (2013)). **Supplemental Table S3.** Coordinates of autosomal and mitochondrial SNPs in lion SNP panel. **Supplemental Table S4.** Genotype calls for all individuals included for genotyping with the SNP panel (125 autosomal + 14 mtDNA SNPs). **Supplemental Table S5.** Assignment values from SNPpanel SNPs. STRUCTURE analysis was done on 125 nuclear SNPs with the complete dataset (N = 211) and a reduced dataset excluding samples with >25% missing data (N=171, '-' for assignment indicates that this sample was excluded). Haplogroups were infered from callings of 14 mtDNA SNPs which were included in the SNP panel (see Bertola et al. (2016) for reference of haplogroups). If 40% or more of the mtDNA SNPs failed, the field for the haplogroup is marked with '?'. Haplogroups marked with '?' after the assignment indicate that 60%-85% of the included mtDNA SNPs were succesfully called. **Supplemental Table S6.** Observed heterozygosity for lions, based on SNP data from either whole genome sequencing (individuals; all SNPs and SNPs covered in 5 or more lions) or based on results of the SNP panel (populations), and comparison with observed heterozygosity based on microsatellite data (populations). Shading indicates the ranking from low heterozygosity (red) to high heterozygosity (green). It must be noted that due to low coverage in the sample from Benin, heterozygosity may be underestimated. For the SNP panel, only results from the same samples which had previously been analyzed for the microsatellites were included. The left scatter plot shows the correlation between observed heterozygosity called based on all SNPs and based on SNPs covered in 5 or more lions. The right scatter plot shows the correlation between observed heterozygosity based on all SNPs and based on the SNP panel data (grey), and observed heterozygosity based on previously published microsatellite data (red). The bar plot shows observed heterozygosity for each dataset and per individual/population. **Supplemental Table S7.** Genbank entries to filter bacterial reads in contaminated lion samples Benin and RSA. **Supplemental Table S8.** Tiger scaffolds from Cho et al. (2013) identified as potentially of Y-chromosomal origin.

## Data Availability

The datasets generated and analysed during the current study are available through NCBI under Bioproject ID PRJNA597374 (accession numbers SRX7438502- SRX7438512) (fastq format), and included within the article and its supplementary information files (SNP panel genotypes).

## Abbreviations

CITES: Convention on International Trade in Endangered Species of Wild Fauna and Flora
DRC: Demoratic Republic of the Congo
EAZA: European Association of Zoos and Aquaria
FIV_Ple_: Feline Immunodeficiency Virus
IUCN: International Union for Conservation of Nature
ML: Maximum Likelihood
mtDNA: Mitochondrial DNA
NGS: Next Generation Sequencing
NP: National Park
nuDNA: Nuclear DNA
PCA: Principal Component Analysis
RSA: Republic of South Africa
SNP: Single Nucleotide Polymorphism
